# Factors affecting acid protease production by *Mucor circinelloides* MG603064.1 through SmF process: characterization and *fromage frais* making

**DOI:** 10.5114/bta.2023.132770

**Published:** 2023-12-21

**Authors:** Souhila Bensmail, Khaled Boudjema, Fethia Naimi-Fazouane, Samira Bensmail, Djamila Djouahra-Fahem, Fatiha Ferhoum, Nassima Bourfis

**Affiliations:** 1 Department of Biology, Faculty of Nature and Life Sciences and Earth Sciences, Akli Mohand Oulhadj University, Bouira, Algeria; 2 Research Laboratory of Food Technology, M’hamed Bougara University, Boumerdes, Algeria; 3 Laboratory of Valorization and Conservation of Biological Resources, M’hamed Bougara University, Boumerdes, Algeria; 4 Department of Agricultural Sciences, Faculty of Nature and Life Sciences and Earth Sciences, Akli Mohand Oulhadj University, Bouira, Algeria

**Keywords:** *Mucor circinelloides*, acid protease, submerged fermentation, milk-clotting activity, *fromage frais*

## Abstract

The exploitation of food industry wastes and their conversion into value-added products present a promising and continuously growing field, given the diversity of elaborated wastes. The current work aimed to utilize sweet cheese whey as a growth medium for acid protease production by a local fungus strain. The biochemical and physicochemical properties of the cheese whey, such as pH, conductivity, chemical oxygen demand, biological oxygen demand (BOD_5_), total nitrogen and protein contents, and mineral salts, were assessed using various analytical methods. The effect of certain parameters on acid protease production by *Mucor circinelloides* MG603064.1 through the SmF process was investigated using the conventional design method “One factor at a time”. Subsequent to characterization, the crude extract was used in a trial to create *fromage frais*, compared to the commercial rennin CHY-MAX^®^ Powder Extra. Cheese whey characterization revealed its richness in total nitrogen (1.044 ± 0.044 g/l), protein content (6.52 ± 0.04 g/l), and principal mineral salts: calcium (1.637 ± 0.037 g/l), phosphorus (1.173 ± 0.023 g/l), and chloride (1.66 ± 0.09 g/l). The optimal values of the SmF process for acid protease production, such as the inoculum size, beef extract, and KH_2_PO_4_ supplements, the initial pH of cheese whey, and incubation temperature were, respectively, 11% (v/v), 0.4% (w/v), 0.5% (w/v), 5.5, and 30°C. Under these conditions, the lowest milk-clotting time of 290 s was achieved, representing an 18.41-fold increase compared to the initial step using the unoptimized medium. The enzyme showed maximum milk-clotting activity at pH 5, a temperature of 60°C, and in the presence of 0.025 M of CaCl_2_. The enzyme activity also significantly improved with sonication (35 kHz) for 10 min. The crude extract of *M. circinelloides* ensured the production of fresh cheese samples with characteristics roughly similar to those obtained by the control (CHY-MAX^®^ rennin). The acid protease of *M. circinelloides* could successfully substitute the conventional rennin in the manufacture of fresh cheese.

## Introduction

Submerged fermentation (SmF) and/or solid-state fermentation (SSF) are employed to produce industrial microbial enzymes. Despite their respective benefits, both technologies present their limitations. Indeed, SmF is often utilized to produce industrial microbial enzymes due to its ease of handling at large-scale operations when compared to SSF (Singhania et al., 2015; Niyonzima et al., 2020).

Proteases represent the largest group of commercially available enzymes worldwide, accounting for 60% of the industrial enzymes market due to their wide range of applications (Liu and Kokare, 2017; Martínez-Medina et al., 2019). They constitute a broad and complex group of enzymes that exhibit differences in various properties, such as substrate specificity, nature of their active sites, catalytic mechanism, optimum pH, optimum temperature, and stability profile (Martínez-Medina et al., 2019).

Microbial proteases are predominantly produced by SmF, which is responsible for nearly 90% of the production of all industrial enzymes. This technique involves cultivating microorganisms in a liquid nutrient broth containing approximately 50 g/l of solutes with a water content of 95%. The advantages of SmF include ease of controlling the process and recovery of extracellular enzymes (Gimenes et al., 2019; Martínez-Medina et al., 2019). The primary factors influencing product production through this bioprocess include the type of fermenter, inoculum size, culture medium composition, dissolved oxygen, and the temperature and pH of the growth medium (Martínez-Medina et al., 2019).

Cheese whey, a by-product of the dairy industry, refers to the liquid stream derived from the transformation of milk into cheese (Lappa et al., 2019). The global whey production is estimated to be around 180 to 200 × 10^6^ tonnes/year (Lappa et al., 2019; Buchanan et al., 2023), of which about 50% is directly disposed of since it is neither processed nor valorized (Bosco et al., 2018; Lappa et al., 2019).

Considered the most important pollutant of the dairy industry, cheese whey is associated with serious environmental hazards due to its very high biological oxygen demand of 27–60 g/l and chemical oxygen demand of 50–70 g/l. This is induced by its composition, which retains 55% of total milk nutrients (Lappa et al., 2019; Zotta et al., 2020).

The nature of whey as a rich lactose, mineral, and protein source provides suitable conditions for the development of microorganisms. This renders the valorization of waste whey to produce value-added compounds an attractive, cost-reduced alternative (Buchanan et al., 2023). Contrastingly, in Algeria, most cheese industries discharge whey – amounting to 13 860 tons/year – into the environment without implementing sustainable treatments. This poses a serious pollution problem due to its high organic load (Acem et al., 2018; Ghobrini et al., 2019). Since whey production is nine times that of cheese production and experiences a 1–2% annual growth rate (Lappa et al., 2019; Buchanan et al., 2023), valorizing this waste is crucial for mitigating severe economic and environmental management issues.

Therefore, the aim of the present study was to enhance the value of cheese whey obtained from the local dairy industry by producing a milk-clotting enzyme (MCE) using the fungal strain *Mucor circinelloides* MG603064.1 through submerged fermentation and to optimize various parameters of the process.

## Materials and methods

### Biochemical composition and physicochemical analysis of cheese whey

Sweet cheese whey (SCW) from the Edam cheese production process was collected from the dairy and cheese factory of Boudouaou (LFB, Boumerdes, Algeria). As part of the physicochemical analyses of SCW, conductivity and pH were measured at 20°C using a Jenway 4520 conductivity meter (Dunmow, UK) and a Hanna Model-209 pH meter (Canada), respectively. Total nitrogen and protein contents were estimated using the Kjeldahl method (Audigié et al., 1984).

The chemical oxygen demand (COD) and the biological oxygen demand (BOD_5_) of SCW were determined following the protocol described by Prorot (2008), employing a spectrophotometer (UV-1800 Shimadzu Corporation, Kyoto, Japan) at 585 nm for COD, and the WTW OxiTop^®^IS system (Germany) for BOD determination.

Atomic absorption spectroscopy (AAS) (Philips AAS model PU 9200) was used to quantify minerals such as Na, Ca, K, Zn, and Mg. For sample preparation, ashes (0.5 g) resulting from the mineralization of whey (5 ml incinerated in a muffle furnace at 530°C for 5 h) were mixed with 3 ml of demineralized water and 3 ml of concentrated HCl, then heated – without exceeding 250°C – until the ashes completely dissolved. The final volume was adjusted to 100 ml with demineralized water and used for mineral salt content determination by AAS. The concentration of each element (in g/l) was deduced from its appropriate calibration curve.

The colorimetric method described by Colombie (2005) was applied to determine the amount of phosphorus in the cheese whey. Four milliliters of the sample or KH_2_PO_4_ solution as the standard (0, 1, 1.5, 2, 2.5, 3, and 5 mg/ml) were added to 0.8 ml of ammonium molybdate reagent. The resulting mixture was vigorously stirred and then allowed to stand for 15 min. Subsequently, the absorbance was measured using a spectrophotometer at 720 nm against a blank, which was prepared using distilled water instead of the sample and was treated in the same manner.

The concentration of chloride in SCW was assayed according to the procedure established by Charpentier-Volhard (AFNOR, 1980). Twenty milliliters of the product to be analyzed, 2 ml of 15% (w/v) potassium hexacyanoferrate II, and 2 ml of zinc acetate solution of 30% (w/v) were introduced into a 100 ml flask. After stirring, the volume of the mixture was brought up to 100 ml with distilled water, followed by filtration through Whatman^®^ filter paper n° 3. Twenty milliliters of the filtrate were then added to 5 ml of silver nitrate (0.1 N), 1 ml of concentrated nitric acid, and 2 ml of 38% (w/v) ammonium iron (III) sulfate. After homogenization, this mixture was titrated with a potassium thiocyanate solution (0.1 N) until an orange–red color appeared (V). The chloride content, given in g of NaCl/l, was calculated according to the following formula:


Chloride content[g/l]=0.585×(5−V) (1)

### Microorganism and inoculum preparation

*M. circinelloides* (von Tieghem) MG603064.1 was previously isolated from the soil of an agricultural area (Tipaza, Algeria), and identified based on both phenotypic and molecular analysis, specifically through the amplification and sequencing of the ITS1-5.8S rDNA-ITS2 region (Bensmail et al., 2020). The strain was stored on Potato-Dextrose Agar (PDA) slants at 4°C and transferred at intervals of 2 or 3 months.

For the preparation of the inoculum, *M. circinelloides* was cultured on PDA medium in Petri dishes at 28± 2°C for 5 days to ensure complete sporulation. Spore suspensions for inoculation were prepared by adding a sterile solution of Tween-80 (0.1%, v/v) and gently scraping the plates. After filtering through Macherey-Nagel filter paper n° 4 (Macherey-Nagel GmbH & Co. KG, Düren, Germany), 1 ml of the resultant suspension, containing 6.2 × 10^7^ spores/ml, was utilized as the inoculum for the fermentation process.

### Optimization of MCE production

The strategy adopted during the optimization of fermentation parameters is the *One-factor-at-a-time* design, generally used to optimize the growth medium in SmF. In this approach, all variables except one are kept constant while determining the optimal level of the testing variable (Benluvankar et al., 2016). This strategy evaluates the individual effect of one factor before proceeding to the next.

The fermentation was conducted in 250 ml Erlenmeyer flasks containing 50 ml of SCW, adjusted to pH 4 with 1 N HCl. These flasks were then autoclaved at 121°C for 20 min and allowed to cool to room temperature. After cooling, the medium was inoculated with 1 ml of the spore suspension (6.2 × 10^7^ spores/ml), as previously prepared, and incubated at 35°C for 72 h at 120 rpm in a rotary shaker (MaxQ 4000, Model 4331, Thermo ScientificTM, Marietta, USA).

To optimize acid protease production, the fermentation was carried out under specific conditions, including different inoculum sizes (5.5, 8, 11, 14% v/v), various concentrations of beef extract (0.2, 0.5, 1, 1.5, 2% w/v), KH_2_PO_4_ (0.2, 0.4, 0.6, 0.8% w/v), different medium pH levels (3, 3.5, 4, 4.5, 5, 5.5, 6), and incubation temperatures (25, 30, 35, 40, 45°C). Conversely, other conditions such as stirring speed and incubation time were held constant (120 rpm and 72 h, respectively) throughout all the optimization experiments.

At the conclusion of each fermentation process, fungal mycelium was separated by centrifugation at 4000 rpm for 10 min at 4°C using a HERMLE refrigerated centrifuge model Z300K from Germany. The resulting supernatant was collected and used as the source of extracellular enzymes (crude extract).

### Milk-clotting activity

The milk-clotting activity (MCA) of the crude enzymatic extracts was determined following the method described by Arima et al. (1970). One milliliter of the enzyme solution (V_2_) was mixed with 10 ml of a skim milk powder solution (10% w/v, pH 6.4) containing 10 mM CaCl_2_ (V_1_). The substrate was allowed to stand for 1 h at 27°C, followed by incubation at 35°C for 15 min, before adding the crude extract. The time (T in seconds) elapsed between mixing the reagents and the initial appearance of milk clots was measured. The MCA, expressed in Soxhlet Units (SU), was determined using the following equation:


$$\text{MCA}\left[\text{SU}/\text{ml}\right]=\frac{2400\times V_{1}}{\text{T}\times V_{2}}$$ (2)

### Protease activity

The proteolytic activity (PA) of the crude extracts was assessed using a modified Anson (1938) method. To do this, 0.5 ml of the enzyme extract was mixed with 2.5 ml of a 2.5% (w/v) casein solution prepared in 0.1 M citrate/sodium phosphate buffer at pH 5.2. The reaction mixture was incubated at 40°C for 30 min and then halted by adding 5 ml of a 0.245 M trichloroacetic acid (TCA) solution. The resulting precipitate was removed by filtration through Whatman filter paper. To 1 ml of the filtrate, 2.5 ml of 0.188 M sodium carbonate and 0.5 ml of a 10-fold diluted Folin Ciocalteau reagent were added. The blue color that developed after incubation for 30 min at room temperature was measured at 750 nm (UV-1800 Shimadzu Corporation spectrophotometer, Kyoto, Japan). A blank test, in which the crude extract was added after the addition of TCA, was carried out for each assay.

Aromatic amino acid L-Tyrosine (at concentrations of 0, 20, 40, 60, 80, and 100 μg/ml) served as the standard. The proteolytic activity, expressed in U, is defined as “the amount of enzyme that releases the equivalent of 1 μg of tyrosine per min per ml of enzyme extract under standard assay conditions.”

### Characterization of enzyme in the crude extract

For the determination of the optimum temperature for MCA in the crude extract, a standard assay was conducted at various milk temperatures (25, 30, 35, 40, 45, 50, 55, 60, 65, and 70°C). The temperature at which maximum MCA was observed was designated as 100%, and the relative activities at other test temperatures were calculated.

To determine the optimum pH for MCA in the crude extract, the standard substrate (rehydrated skim milk) was adjusted to pH levels of 5, 5.5, 6, 6.5, 7, and 7.5. Subsequently, MCA was measured under the standard conditions at 35°C. The maximum activity obtained was considered as 100%, and the relative activities at other pH levels were calculated.

The effect of CaCl_2_ concentration (5, 10, 15, 20, 25, 30, 35, 40, 45, and 50 mM) added to the reaction medium on the MCA of the crude extract was investigated under standard assay conditions (35°C and pH 6.4).

To study the impact of ultrasound cavitation on the milk-clotting protease of *M. circinelloides*, the MCA of the crude extract was assessed before and after treatment. The crude extract was subjected to ultrasound at a constant frequency of 35 kHz for varying durations (1, 3, 5, 10, 15, 30, and 60 min). The ultrasonic bath temperature was maintained at 10 ± 2°C during the experiment using the Sonorex Digitec from BANDELIN Electronic in Berlin, Germany.

### Production of fromage frais

The crude enzymatic extract of *M. circinelloides* was applied in a trial for fresh cheese production using cow milk and compared to the commercial rennet CHY-MAX^®^.

The process for manufacturing fresh cheese involved treating cow’s milk with coagulating enzymes and a starter culture of lactic acid bacteria (LAB). Six liters of raw cow’s milk, sourced from the Boumerdes region in Algeria, were used for each batch. The milk’s initial characteristics were as follows: pH 6.75, fat content 32 g/l, total solids 116.7 g/l, defatted dry extract 83.68 g/l, titratable acidity 1.5 g/l, and density 1.029 g/ml, with complete absence of antibiotics for each essay.

The milk was pasteurized by heating it to 90°C for 15 s, then cooled to 38 ± 2°C and maintained at this temperature in a water bath. Starter cultures (0.05 g/l) (*Lactococcus lactis* subsp. *cremoris*; *Lactococcus lactis* subsp. *lactis* from Chr. Hansen A/S-Improving Food and Health, 2970 Hoersholm, Denmark) and CaCl_2_ (0.25 g/l) were added to the milk, which was thoroughly mixed for 10 min and allowed to ripen until the pH reached 6.4.

At this point, either commercial rennet CHY-MAX^®^ Powder Extra (0.075 g/l of milk) (2235 IMCU/g, Chr. Hansen A/S-Improving Food and Health, 2970 Hoersholm, Denmark) or the crude enzymatic extract of *M. circinelloides* obtained under optimum conditions (2.7 ml/l of milk) was added separately to initiate the coagulation of casein. The milk was stirred to ensure even distribution of the enzymes and left undisturbed for 30–40 min until a firm coagulum formed.

Afterward, the resulting curds were manually cut using cutters, gently stirred, and then allowed to settle for 10 min. The whey was drained for approximately 16 h after transferring the curd/whey mixture to perforated molds lined with cheesecloth. The cheeses were periodically turned into the molds until the pH dropped to 4.4. At this stage, they were dry salted at room temperature, incorporated into the cheese mass. Finally, the cheeses were packed in cellophane paper and stored at 4°C.

### Analysis of the quality of cheese

The chemical composition of the cheese samples was evaluated using standard analyses: dry matter and water content (moisture) were estimated by oven-drying the cheese at 102°C until a constant weight was achieved (ISO 5534-IDF 4: 2004). The fat content was determined using the Van Gulik method (ISO 3433-IDF 222: 2008), and the pH was measured by placing the pH meter electrode in contact with the curd (Ardö and Polychroniadou, 1999).

The microbiological quality of the cheeses after production was evaluated by detecting and enumerating the following germs: total coliforms (Desoxycholate Agar, 37°C, 24 h) (ISO 4832: 2006), fecal coliforms (Desoxycholate Agar, 44°C, 24 h) (NF V08-060-April 2009, AFNOR), *Staphylococcus aureus* (Baird Parker Agar, 37°C, 48 h) (ISO 6888-1: 1999), and *Salmonella* spp. (enrichment in Selenite-cysteine Broth and isolation on Hektoen Agar at 37°C for 72 h) (ISO 6785-IDF 93: 2001). Similarly, the microbiological quality of pasteurized milk was assessed using the same methods detailed above, with counting the total aerobic flora (Plate Count Agar, 72 h, 30°C) (ISO 6610: 1992).

The sensory quality of the prepared products was assessed by trained tasters from the production unit (The Dairy and Cheese Factory of Boudouaou, Algeria). The attributes assessed included the appearance, color, odor, texture, and taste of the cheeses.

### Statistical analysis

The fermentation batches were carried out in duplicate, and the results were reported as means ± standard deviation (SD) based on a minimum of three replicates. The software Excel version 7.0 (Microsoft Corp., USA) was used to calculate data.

To determine the impact of sonication on enzyme activity, statistical significance (whether positive or negative) was assessed at a significance level of *P* < 0.05. This analysis was calculated using JMP^®^ Pro 13.2.1 software (SAS Institute Inc., United States) and involved the Student *t*-test.

## Results and discussion

### Composition and physicochemical characteristics of the cheese whey

In our study, the production of proteolytic enzymes by fungi was found to be influenced by several parameters, with the composition of the culture medium playing a crucial role. Therefore, the initial step in our study was to evaluate the biochemical composition of the cheese whey, which served as the SmF medium for protease production by *M. circinelloides* MG603064.1.

As indicated in Table 1, our results revealed that the cheese whey used for protease production exhibited elevated levels of BOD_5_ and COD, measuring 50.4 ± 0.6 and 75.7 ± 0.4 g of O_2_/l, respectively. This corresponded to a COD/BOD_5_ ratio of 1.50, indicating a moderate level of biodegradability for this waste. It is important to note that this by-product is a significant contributor to environmental pollution, impacting the physicochemical characteristics of soil and reducing aquatic life due to oxygen content (Panesar et al., 2007; Papademas and Kotsaki, 2019). With a COD reaching as high as 100 g/l, direct disposal into surface water is not feasible, necessitating careful consideration in any biological treatment process without previous dilution (Bosco et al., 2018).

**Table 1:** Physicochemical characteristics of the sweet cheese whey

pH	6.17 ± 0.020
Conductivity [:s/cm]	7.25 ± 0.020
Composition [g/l]	
Total nitrogen	1.044 ± 0.044
Total protein	06.52 ± 0.040
Chemical Oxygen Demand (COD)	75.70 ± 0.400
Biological Oxygen Demand (BOD_5_)	50.40 ± 0.600
Mineral salts:	
Sodium (Na)	0.737 ± 0.001
Calcium (Ca)	1.637 ± 0.037
Potassium (K)	0.783 ± 0.003
Zinc (Zn)	0.003 ± 0.000
Magnesium (Mg)	0.226 ± 0.012
Phosphorus (P)	1.173 ± 0.023
Chloride (Cl)	1.660 ± 0.090

Furthermore, our analysis revealed that cheese whey contained notable levels of nitrogen (1.044 ± 0.044 g/l) and proteins (6.52 ± 0.04 g/l). Additionally, the recorded conductivity was 7.25 ± 0.02 μs/cm, indicating the abundance of mineral salts in this by-product. These findings were further corroborated by AAS, which detected trace amounts of mineral elements such as Na, Ca, K, Zn, Mg, and P. Similar data were reported by Papademas and Kotsaki (2019) for parameters such as pH (5.9–6.4), COD (50–80 g/l), BOD (40–60 g/l), total protein (6.5–6.6 g/l), phosphorus (1–3 g/l), and chloride content (1.1 g/l) in sweet cheese whey. However, it is worth noting that the calcium concentration obtained in our study (1.637 ± 0.037 g/l) was higher than the values cited by Panesar et al. (2007) and Papademas and Kotsaki (2019) (0.4–0.6 g/l).

### Optimization of the fermentation parameters

In SmF, the composition of the media plays a significant role in influencing enzyme production by microorganisms. Additionally, environmental factors such as temperature, pH, and incubation time have a substantial impact on microbial metabolism. As a result, optimizing these factors becomes crucial in promoting, stimulating, and enhancing the production of proteases (Sharma et al., 2017). Indeed, various approaches have been applied to optimize the different factors, both physicochemical and nutritional, that affect the fermentation process for microbial protease production.

During our experimental approach, we chose to adopt the *One-factor-at-a-time* method. This approach allowed us to systematically study the effect of chosen parameters in the SmF process and seek improvements in protease production by *M. circinelloides*.

#### Effect of inoculum size

The production of enzymes is significantly influenced by the size of the inoculum, which determines the amount of biomass generated during the fermentation process (Amer et al., 2015; Niyonzima et al., 2020).

**Fig. 1:**
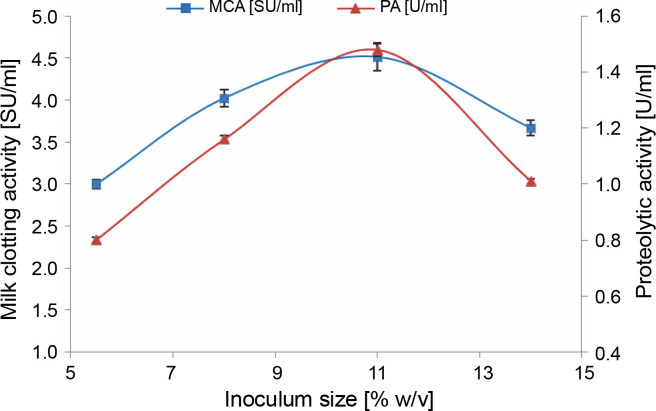
Effect of inoculum size on MCE production by *M. circinelloides* (conditions: initial medium pH = 4, incubation temperature = 35°C, incubation period = 72 h)

We observed that there was a significant increase in milk-clotting protease production by *M. circinelloides* (4.51 ± 0.16 SU/ml) as the inoculum concentration was enhanced, reaching its optimum at 11% (v/v). This increase was attributed to the rapid degradation of the substrate (Fig. 1). Beyond this optimal inoculum size, both the milk-clotting activity and the proteolytic activity of the crude enzymatic extract showed a slight decline. The decrease in enzyme activities when an inoculum level of 14% (v/v) was used can be attributed to the faster growth of the fungus, which led to the depletion of available nutrients and oxygen required for the biomass produced (Amer et al., 2015; Niyonzima et al., 2020). Conversely, lower protease production (3 ± 0.05 and 4.02 ± 0.10 SU/ml) associated with smaller inoculum sizes (5.5 and 8%, v/v) may be linked to the lower resultant biomass, which resulted from a more extended lag phase. This insufficient biomass failed to efficiently hydrolyze nutrients for maximum protease production within the same fermentation time.

It is worth noting that the impact of inoculum size on microbial protease production can vary from one microorganism to another. For example, Zhou et al. (2009) reported that an inoculum size of 15% was optimal for neutral protease production by *Laccocephalum mylittae*, while an inoculum concentration of 2% was found to be ideal for rennin-like acid protease production by *M. pusillus* IHS6 (Daudi et al., 2015). Moreover, optimal MCE production by *Bacillus amyloliquefaciens* strains was observed at different inoculum sizes ranging from 1 to 7% (v/v) in various studies (He et al., 2012; Guleria et al., 2016; Zhang et al., 2019).

#### Effect of beef extract addition

The specific nutritional requirements of microorganisms used in industrial fermentation processes are indeed intricate and diverse, varying not only by the type of microorganism (typically bacteria, molds, and yeast) but also by the specific species and strains involved (Kampen, 2014). This complexity leads to highly specific nutritional requirements.

Among all the mineral nutrients, carbon and nitrogen are required in the highest amounts and often serve as limiting factors for fungal growth and enzyme production (Deacon, 2006; Sharma et al., 2017). The necessity for a particular nitrogen supplement to enhance protease production varies from one microorganism to another. Each microorganism may prefer a distinct source of nitrogen, which can be either simple or complex, mineral or organic.

**Fig. 2:**
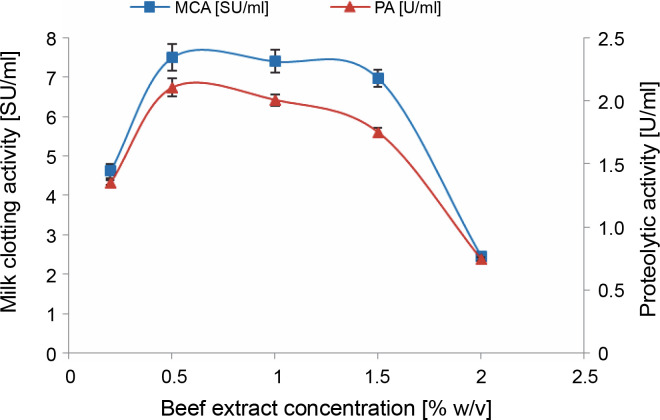
Effect of beef extract concentration on MCE production by *M. circinelloides* (conditions: medium pH = 4, inoculum size = 11% (v/v), incubation temperature = 35°C, incubation period = 72 h)

As depicted in Figure 2, the highest milk-clotting (7.5 ± 0.33 SU/ml) and proteolytic activities (2.1 ± 0.074 U/ml) were achieved when using a 0.5% (w/v) beef extract supplement. It is important to note that these results contrast with those obtained by Daudi et al. (2015), who found that during submerged fermentation, beef extract induced lower protease production by *M. pusillus* IHS6 compared to other organic nitrogen sources such as yeast extract, corn steep liquor, peptone, nutrient broth, meat extract, and casein.

Beef extract serves as a complex organic nitrogen source that offers a wide range of nutrients, including micronutrients and macronutrients, vitamins, growth factors, carbon, and energy (Niyonzima et al., 2020). The incorporation of such nitrogen sources into the fermentation medium reduces the need for microorganisms to synthesize these compounds *de novo* simplifying their metabolic processes (Kampen, 2014). However, it is crucial to carefully consider the concentration of these additives to prevent any adverse effects on the production of the targeted metabolite.

In the case of cheese whey, which already contains nitrogen at a concentration of 1.044 ± 0.044 g/l, the addition of beef extract positively impacted protease production by *M. circinelloides* up to a concentration of 0.5% (w/v) of beef extract. Beyond this point, a phenomenon known as nitrogen metabolic repression occurred, leading to reduced levels of enzymatic activities. This outcome aligns with findings previously presented by Daudi et al. (2015). According to Niyonzima et al. (2020), the repression induced by high amounts of complex organic nitrogen sources can be attributed to their abundance of amino acids (such as glycine and valine) and short peptides, which can inhibit enzyme secretion by microorganisms.

#### Effect of KH_2_PO_4_ supplementation

In many cases, complex or natural media must be supplemented primarily with inorganic nutrients to fulfill the requirements of the fermenting organism (Kampen, 2014). Micronutrients, in particular, play a crucial role in maintaining the osmotic pressure of the production medium, and appropriate concentrations of trace elements are reported to be essential for proper substrate utilization by microbes (Sattar et al., 2019).

**Fig. 3:**
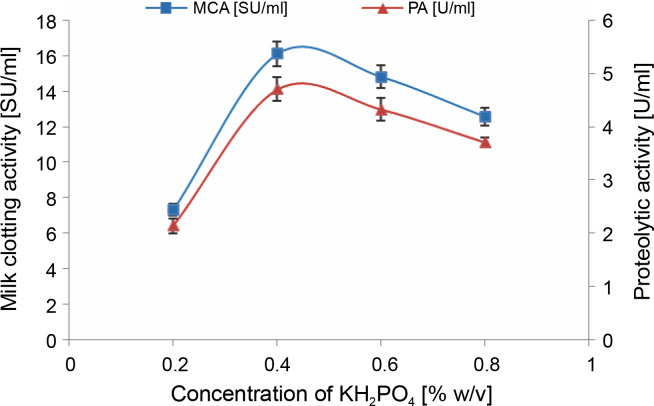
Effect of KH_2_PO_4_ concentration on MCE production by *M. circinelloides* (conditions: medium pH = 4, inoculum size = 11% (v/v), beef extract concentration = 0.5% (w/v), incubation temperature = 35°C, incubation period = 72 h)

As illustrated in Figure 3, the addition of 0.4% (w/v) KH_2_PO_4_ supplement to cheese whey proved to be optimal, resulting in the highest MCA of 16.1 ± 0.7 SU/ml and PA of 4.7 ± 0.22 U/ml. This represented an increase of over 2-fold compared to the previous step. Similar findings were reported by Wehaidy et al. (2020), who studied the production of MCE by *B. subtilis* MK775302 and found an optimum concentration of 0.3% (w/v) KH_2_PO_4_. However, for MCE production by *M. mucedo* KP736529, Abou Ayana et al. (2015) determined that a lower concentration of KH_2_PO_4_ (0.132%, w/v) was optimal.

Potassium and phosphorus are typically required in relatively large quantities to support fungal growth and metabolite production and should be included as salts in culture media (Kampen, 2014). These elements are categorized as macronutrients and are provided at millimolar concentrations (Walker and White, 2018). The specific supply of potassium and phosphorus depends on the composition of the broth, the organism’s requirements, and the nature of the desired product (Singh et al., 2017). Phosphate is crucial for synthesizing phospholipids present in microbial cell membranes, as well as for nucleic acids, adenosine triphosphate (ATP), glycophosphates, and polyphosphates production (Deacon, 2006; Singh et al., 2017; Walker and White, 2018). Potassium, on the other hand, is necessary as an enzyme cofactor and for osmoregulation (Walker and White, 2018).

Kampen (2014) notes that bacteria and fungi can generally tolerate up to 5 g/l of potassium phosphate, a conclusion our findings support. We observed a decrease in MCE activities (14.8 ± 0.64 and 12.56 ± 0.5 SU/ml) when KH_2_PO_4_ was added to cheese whey at a concentration exceeding 4 g/l. In a hypertonic state (high concentration of salts in the fermentation medium), water molecules flow out of the microbial cell walls through osmosis, causing the cell wall to shrink and, ultimately, decreasing enzyme production. Conversely, in hypotonic conditions, water penetrates the microbial cells, causing them to swell or burst (Sattar et al., 2019).

#### Effect of initial pH

Among the physicochemical parameters, the initial pH of the growth and fermentation medium critically influences by inducing morphological changes in microbes and affecting enzyme secretion (Manivannan and Kathiresan, 2007). It also establishes the nature of the end product – acidic, alkaline, or neutral – which subsequently contributes to its commercial application in the industry (Sattar et al., 2019).

**Fig. 4:**
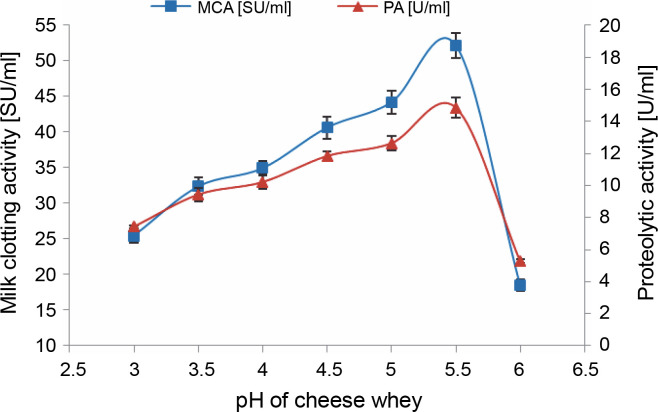
Effect of SCW pH on MCE production by *M. circinelloides* (conditions: inoculum size = 11% (v/v), beef extract concentration = 0.5% (w/v), KH_2_PO_4_ concentration = 0.4% (w/v), incubation temperature = 35°C, incubation period = 72 h)

In this phase, MCE production by *M. circinelloides* was achieved under the optimal conditions previously established, but with cheese whey pH varying from 3 to 6. Protease production gradually increased within the chosen pH range, peaking at pH 5.5 (52.156 ± 1.764 SU/ml) (Fig. 4). Beyond this, the enzymatic activities decreased by approximately 64.5% at pH 6, owing to the impact of pH on enzyme stability in the medium.

Likewise, a lower pH value of 5.5 has been demonstrated as the optimum initial pH for the production of rennin-like acid protease (56 U/ml) by *M. pusillus* IHS6 (Daudi et al., 2015). However, our findings stand in contrast to those presented by Amer et al. (2015), where maximum protease production (35 SU/ml) using *M. miehei* NRRL 3420, was achieved at a pH value of 4 on the same substrate. In another instance, the highest MCA of *Fusarium subglutinans* protease (78.43 SU/mg dry biomass) was obtained at an initial medium pH of 6 (Ghareib et al., 2001).

The culture medium pH potentially impacts all enzymatic processes and the transport of various components across the cell membrane, thereby supporting cell growth and product formation (Sharma et al., 2017; Niyonzima et al., 2020). Shifts in pH influence growth by affecting the net charge on membrane proteins and cell wall enzymes, the ionic states of different components in the medium, and the degree of dissociation of mineral salts, all of which have potential consequences for nutrient uptake and membrane permeability (Deacon, 2006; Kampen, 2014).

Changes in pH may induce enzyme denaturation, leading to a loss of catalytic activity. This shift might also involve modifications in the ionic state of the substrate, potentially resulting in the creation of charged particles that may not align with the ionic active sites of the enzyme. Under these circumstances, enzyme-substrate complexes are not formed, rendering the substrate unavailable to the microorganisms. Consequently, each enzyme functions optimally within a specific pH range unique to it, and its activities are reduced with a significant decrease or increase in that value (Mukhtar and Ul-Haq, 2009).

#### Effect of temperature

The temperature of the culture is a pivotal parameter for attaining maximum cell growth and enzyme production. It necessitates meticulous control and may vary among different organisms (Sharma et al., 2017). According to Kumar et al. (2008), fungi are predominantly mesophilic, with an optimal temperature for protease production ranging from 20 to 35°C.

**Fig. 5:**
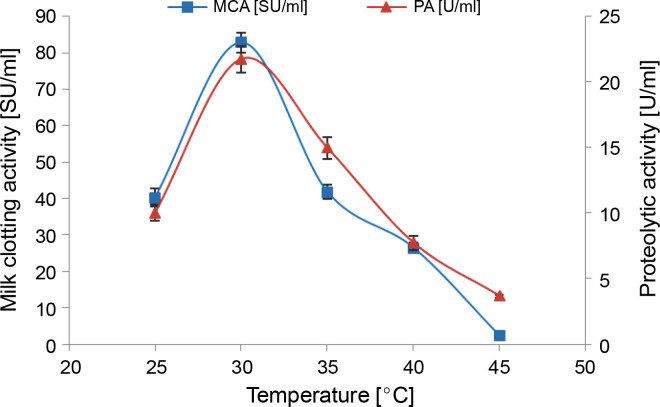
Effect of incubation temperature on MCE production by *M. circinelloides* (conditions: medium pH = 5.5, inoculum size = 11% (v/v), beef extract concentration = 0.5% (w/v), KH_2_PO_4_ concentration = 0.4% (w/v), incubation period = 72 h)

As depicted in Figure 5, temperature positively influenced protease production. The enzyme activities, MCA and PA, peaked after 3 days of incubation at 30°C (82.76 ± 2.85 SU/ml and 21.74 ± 1.02 U/ml, respectively). This value aligns well with the temperatures reported for maximum milk-clotting proteases production by various fungal and bacterial strains: *F. subglutinans* (Ghareib et al., 2001), *Amylomyces rouxii* (Yu and Chou, 2005), *M. pusillus* (Daudi et al., 2015), and *B. amyloliquefaciens* SP1 (Guleria et al., 2016) in liquid medium fermentation.

The enzymatic activities of the crude extract produced by *M. circinelloides* MG603064.1 decreased above an incubation temperature of 30°C. The MCA of the crude extract was completely abolished at 45°C, with an absence of milk coagulation even after 2 h. However, its proteolytic activity lingered at around 37% of that achieved at the optimal temperature (30°C).

According to Daudi et al. (2015), such results can be elucidated by the effect of temperature on the physiological processes of microorganisms and the sensitivity of enzymes. Indeed, a rise in this physical parameter, within certain limits, enhances the activity of enzymes, but extremely high temperatures prove destructive. An enzyme loses its catalytic properties at elevated temperatures due to the stretching and eventual breaking of weak hydrogen bonds within the enzyme structure.

#### Characterization of the crude extract

Numerous factors influence the coagulation of milk by proteases, including temperature, pH, Ca^2+^ concentration, and the concentrations of enzyme and casein, all of which can impact both the primary and secondary stages of the milk-curdling process. Varying the first three factors and observing their influence on milk clotting time facilitate the determination of the optimal conditions of activity for the crude extract of *M. circinelloides*.

#### Optimum temperature

With the elevation in milk temperature, the MCA of *M. circinelloides* crude extract gradually increased up to a limit (60°C), beyond which the emergence of casein flakes was notably delayed. The maximum MCA of the enzyme extract (469.16 ± 6.23 SU/ml) was obtained at an elevated temperature of 60°C, which is proximate to the thermal inhibition temperature of the enzyme (Table 2). At 50°C, the MCA represented only 43% of the maximum activity, which was 201.07 ± 2.77 SU/ml. A mere 10°C increase in this temperature led to the highest activity, which rapidly declined (a loss of 84%) when the milk temperature was increased by an additional 5°C, and the enzyme became inactive at 70°C.

**Table 2:** Influence of some physicochemical factors on the MCA of *M. circinelloides* crude extract

	MCA [SU/ml]	Relative MCA [%]
Temperature [°C]		
25	32.46 ± 0.121	5
30	40.212 ± 0.132	8.571
35	82.80 ± 1.36	17.65
40	108.27 ± 1.20	23.077
45	140.75 ± 1.60	30
50	201.07 ± 2.77	42.86
55	281.50 ± 3.80	60
**60**	**469.16 ± 6.23**	**100**
65	74.13 ± 1.30	15.80
70	0	0
pH of the milk		
5.0	**443.51 ± 3.12**	**100**
5.5	275.31 ± 2.00	62.075
6.0	178.513 ± 0.70	42.26
6.5	82.76 ± 1.40	18.66
7.0	1.86 ± 0.045	0.42
7.5	0	0
CaCl_2_ concentration [mM]		
5	57.40 ± 0.97	45.447
10	82.76 ± 1.40	65.53
15	99.413 ± 1.83	78.712
20	109.80 ± 2.30	86.93
**25**	**126.30 ± 3.85**	**100**
30	107.47 ± 2.12	85.093
35	93.462 ± 1.70	740
40	83.43 ± 1.50	66.06
45	74.90 ± 1.00	59.303
50	58.40 ± 0.80	46.236

Similarly, Sun et al. (2014), da Silva et al. (2019), and Mamo et al. (2022) have reported the maximum MCA of protease produced by *R. microsporus* var. *rhizopodiformis*, *R. microsporus*, and *A. oryzae* DRDFS13, respectively, at 60°C. However, other milk-clotting proteases exhibited maximum activities at varying temperatures: 45°C for the protease from *A. niger* FFB1 (Fazouane et al., 2010) and *B. subtilis* MTCC 10422 (Narwal et al., 2016), 50°C for proteases produced by *Rhizomucor miehei* (Celebi et al., 2016) and *B. methanolicus* LB-1 (Li et al., 2019), 65°C for *Thermomucor indicae* seudaticae (Silva et al., 2013), and 70°C for the protease from *B. subtilis* MK775302 (Wehaidy et al., 2020).

The enzymatic coagulation of casein micelles is a highly temperature-dependent process, and bovine milk does not coagulate below approximately 18°C unless the Ca^2+^ concentration is increased (Fox et al., 2017). The optimum temperature for the coagulation of milk by calf rennet at pH 6.6 is 45–48°C. Nevertheless, during cheesemaking, milk is typically set at 30°C due to the presence of starter cultures (McSweeney, 2007; Fox et al., 2017).

#### Optimum pH

The impact of pH on the MCA of *M. circinelloides* crude extract was assessed in the pH range 5–8, wherein a pH value < 5 induced curdling of the milk even without the addition of extracts. The obtained results (Table 2) demonstrated that the enzyme of *M. circinelloides* MG603064.1 is an acid protease, with the maximum activity (443.51 ± 3.12 SU/ml) observed at pH 5; exceeding this value, the enzymatic activity diminished significantly. The enzyme in the crude extract of *M. circinelloides* lost 38% of its activity at pH 5.5, which further progressively decreased with a subsequent increase in pH until it was inactivated at pH 7, due to enzyme denaturation.

According to Fernandez-Lahore et al. (1999), fungal proteinases are characterized by an optimum pH between 2.5 and 5.5. Several milk-clotting enzymes (aspartic proteases) demonstrated the highest MCA at or very close to the optimal pH discovered in our study (Sun et al., 2014; Celebi et al., 2016; da Silva et al., 2019; Wehaidy et al., 2020; Mamo et al., 2022). This elevated activity of milk-clotting enzymes in acidic pH is highly advantageous in industrial applications (Wehaidy et al., 2020), especially for cheese production (Mamo et al., 2022). The optimum pH for the first stage of rennet action in milk is ~6 at 4 or 30°C (Fox et al., 2017).

The effect of pH is predominantly crucial in the first (enzymatic) stage of rennet coagulation. As the pH of the milk diminishes, the enzyme operates in a more optimal environment for its activity, thereby accelerating the reaction. A subtle impact on the second stage of coagulation also happens as bringing the pH closer to the isoelectric point of the caseins reduces the repulsive charge on the micelles and facilitates aggregation (Fox et al., 2015).

#### Effect of CaCl _2_ concentration

Calcium is recognized as a facilitator for coagulation by establishing isoelectric conditions and functioning as an ion bridge between the phosphate portions of casein micelles (Silva et al., 2013; Sun et al., 2014). In this phase, the impact of varied CaCl_2_ concentrations used to formulate skimmed milk was examined. The coagulation rate of the milk was subsequently monitored at 35°C and a pH of 6.4.

As indicated in Table 2, elevating the calcium content of the milk results in a reduction in coagulation time. The briefest clotting time (190 s) was achieved with a concentration of 0.025 M. Beyond this concentration, the MCA of *M. circinelloides* crude extract experienced a mild decline (107.47 ± 2.12 SU/ml), potentially due to an overly robust increase in the ionic strength of the medium (milk) or the saturation of casein micelles’ carboxyl groups with Ca^2+^ ions. This would augment the net positive charge on the casein and diminish its predisposition to aggregation (McSweeney, 2007; Silva et al., 2013). These results are comparable to those reported for other milk-clotting proteases produced by *B. amyloliquefaciens* D4 (He et al., 2011) and *B. methanolicus* LB-1 (Li et al., 2019) that displayed maximum MCA at the identical CaCl_2_ concentration (0.025 M).

Microbial coagulants from *B. subtilis* MTCC 10422 (Narwal et al., 2016), *T. indicae-seudaticae* (Silva et al., 2013), and *Rhizopus microsporus* var. *rhizopodiformis* (Sun et al., 2014) exhibited a similar profile of calcium concentration effect as *M. circinelloides* protease, with peak activity at 0.03 M and 0.04 M, respectively.

According to the latest study published by Mamo et al. (2022), the maximal MCA of *A. oryzae* DRDFS13 acidic protease was detected at 0.1 M of CaCl_2_. Nevertheless, an excess of this salt may induce the formation of a bitter taste and diminish the meltability of cheese (Fox and Guinee, 2013; Silva et al., 2013).

Calcium serves a pivotal role in the coagulation of milk by rennet and in the subsequent handling of the coagulum. Hence, it is common practice to add 0.01% CaCl_2_ to cheese milk (Fox and Guinee, 2013). A heightened Ca^2+^ level in milk hastens coagulation. The influence of Ca^2+^ primarily pertains to the second stage of rennet coagulation since it is crucial for the aggregation of micelles. Although it has a marginal (indirect) effect on the first stage, the addition of calcium to milk alters its salt balance by precipitating colloidal calcium phosphate and generating H^+^, which lowers the pH (McSweeney, 2007).

#### Effect of sonication

**Fig. 6:**
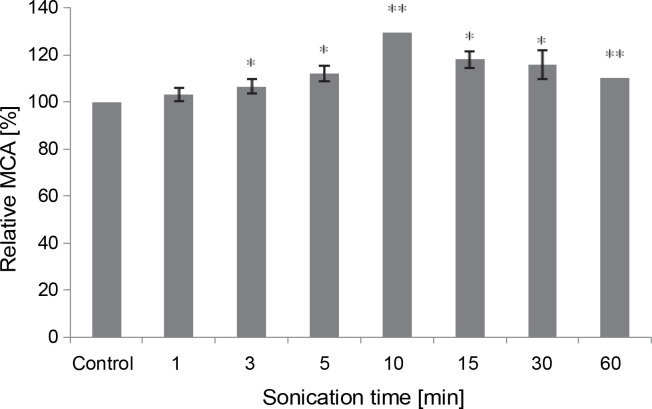
Influence of the sonication time on MCA of *M. circinelloides* crude extract (*P < 0.05, **P < 0.01)

All sonication times tested in the assay positively impacted the MCA of *M. circinelloides* crude extract, compared to the control (Fig. 6). The curdling activity improved gradually and progressively until it peaked after 10 min of contact (107.62 ± 0.0 SU/ml), reflecting an activation percentage of 30%. Subsequently, the MCA decreased to 91.04 SU/ml but remained 10% higher than the untreated control test even after 60 min of sonication. Our results illuminate the stability of the fungal proteolytic extract from *M. circinelloides* when exposed to ultrasound.

Our findings differ from those previously noted by Raharintsoa et al. (1977, 1978). The milk-clotting enzymes utilized in those studies – chymosin, pepsin, and fungal proteases from *M. miehei*, *M. pusillus*, and *Endothia parasitica* – exhibited varying sensitivities to denaturation by ultrasonic cavitation. The authors demonstrated that fungal enzymes were the most resistant, with ultrasound not significantly reducing their MCA even after several minutes of treatment (1 h). However, for animal proteases (chymosin and pepsin), the decrease in such activity (MCA) heightened with irradiation time and factor of dilution (Raharintsoa et al., 1977, 1978).

### Making of fromage frais

*Fromage frais* is a fresh, non-ripened acid-curd cheese that can be consumed immediately or shortly after production. It boasts a very soft consistency and is regarded as a visco-elastic liquid rather than a solid. Characterized by a curd that is notably moist and a fresh, subdued flavor (Schulz-Collins and Senge, 2004; Goddik, 2012; Fox and Guinee, 2013), the nutritional quality of this cheese type is paramount as a protein concentrate (4–10.2/100 g) and a source of calcium (111–127 mg/100 g) (Fox and Guinee, 2013; Cesbron-Lavau et al., 2016). While it can be made without rennet or another coagulating enzyme, rennet is typically added to firm the coagulum, subsequently minimizing the loss of shattered casein curd into the whey (Schulz-Collins and Senge, 2004; Goddik, 2012).

#### Composition and physical properties

The quality of the cheese is influenced by its composition, especially moisture content, NaCl concentration, pH, moisture in nonfat substances, and the percentage of fat in dry matter (FDM) (Fox and Guinee, 2013). Table 3 shows the results obtained regarding the cheese samples’ composition and their physicochemical properties. Data analysis reveals that CHY-MAX^®^ rennin and the crude enzymatic extract of *M. circinelloides* lead to similar cheeses, with slight variations in the chemical composition.

**Table 3:** Physicochemical characteristics of fresh cheeses (*fromage frais*) produced by the crude extract of *M. circinelloides* and CHY-MAX^®^ rennin

Cheese made by	pH	Dry matter [%]	Moisture [%]	Fat [%]	FDM [%]	Yield [%]
CHY-MAX^®^ (Control)	4.65	33.5	66.5	6.67	20	12.10
Crude extract of *M. circinelloides*	4.85	35.6	64.4	6	16.85	10.52

FDM – fat in dry matter

### 1. pH values

The examined fresh cheese samples exhibited a pH of 4.65 and 4.85 for the control and *M. circinelloides* crude extract cheese, respectively, signaling significant lactic fermentation of the products due to these low pH values. Typically, this cheese type boasts a pH value of 4.55 for very low-fat products (0.5%) and 4.65 for creamy fromage frais (containing 8% of fat) (Banks, 1998). Given the fat content of the prepared fresh cheeses (6% and 6.67% for cheese made by the fungal crude extract and CHY-MAX^®^ rennin, respectively), our results align with these values for the control and are slightly higher in the case of the fungal extract.

Most bacteria require a neutral pH value for optimum growth and grow poorly at pH values < 5. The pH of cheese curds after manufacturing typically situates within the range of 4.5 to 5.3. This range is maintained to control bacterial growth in cheese (Fox et al., 2017) and to inhibit the growth of bacterial pathogens during the ripening and preservation processes (Wehaidy et al., 2020).

### 2. Moisture content

According to the results presented in Table 3, the fresh cheese samples were characterized by similar moisture contents, approximately 66.5% for the control (CHY-MAX^®^ rennet) and 64.4% for the cheese produced by the fungal crude extract. Domagała et al. (2020) obtained similar moisture levels (66.46–67.56%) using *Rhizomucor miehei* endopeptidase (Fromase^®^ 2200TL) for fromage frais production from the milk of different cow breeds. According to St-Gelais (2018), the typical moisture content of fromage frais should be around 65–88%.

Pradal (2012) specified that moisture content can vary significantly between different types of cheese. For very fresh cheese, the moisture content is above 80%, but for fromage frais, it ranges between 60–65%.

During the manufacture of all cheeses, a reduction in moisture occurs; the lower it becomes, the harder the cheese is, and the longer it retains its quality (Fox et al., 2017). Due to the high moisture content of fromage frais, its shelf-life is limited to 2–4 weeks at < 8°C. Throughout storage, there should be no appearance of whey, dryness, or graininess, nor should there be bacteriological deterioration, over-acidification, or a bitter flavor (Schulz-Collins and Senge, 2004; Goddik, 2012).

### 3. Dry matter (DM)

*Fromage frais* features a lower DM content (15–20%), diminished levels of fat and protein, and an elevated level of lactose/lactate (2.1–5.5 g/100 g) (Schulz-Collins and Senge, 2004; Cesbron-Lavau et al., 2016). Additionally, the total solid content should be at least 15% when the fat in dry matter (FDM) exceeds 20% and at least 10% when FDM is under 20% (Goddik, 2012). The levels of dry matter achieved during this study – 33.5% and 35.6% for cheese produced by CHY-MAX^®^ rennin and the crude extract of *M. circinelloides*, respectively – do not significantly deviate from the contents reported by Domagała et al. (2020) for fromage frais-type cheese prepared from the milk of three selected cow breeds, where the DM values found were 32.44, 33.26, and 33.54%.

### 4. Fat and FDM contents

Fats and proteins are the main components of fresh cheese. Therefore, any variation in their contents affects the total solid content of the cheese. The fat delivered by fresh milk, existing in the form of a relatively stable emulsion, is more readily retained in the coagulum (Farkye, 2004; St-Gelais, 2018).

Per fromage frais specifications, it should harbor a fat in dry matter (FDM) content ranging from 0 to 58% (Schulz-Collins and Senge, 2004) and typically exhibit a fat content of 6–8% (Banks, 1998). The results illustrated in Table 3 delineate the fat and FDM contents of the cheese samples prepared using commercial rennet (6.67 and 20%, respectively) and *M. circinelloides* crude extract (6 and 16.85%, respectively). These values imply that both kinds of cheese fall into the category of partially skimmed fresh cheese according to CODEX STANDARD 221-2001, which specifies a range of 10% ≤ FDM < 25%. Conversely, the FDM contents reported by Domagala et al. (2020) for the produced fresh cheeses (41.06 ± 1.34%, 43.45 ± 2.2%, and 46.67 ± 1.15%) classified them as medium-fat (25% ≤ FDM < 45%) and all-fat (45% ≤ FDM < 60%) cheeses.

#### Cheese yield

The typical yield of cheese ranges from 9 to 15%, contingent on the chemical composition of the milk, effective recovery of fat and casein in the cheese, losses of milk constituents in the whey due to milk handling and treatment alongside cheesemaking procedures, and the final moisture content of the cheese (Farkye, 2004). According to Pradal (2012), the cheese yield for fromage frais, bearing a moisture content of 60–65%, fluctuates between 14 and 15%.

Results pertaining to cheese yield are presented in Table 3. The data reveal that the application of commercial rennin, CHY-MAX^®^ Powder Extra, culminated in a yield of 12.1%, which was higher than the yield provided by the crude extract of *M. circinelloides* (10.52%). This slight difference in cheese yield (around 1.15%) originates from the expedited proteolysis of caseins, a characteristic intrinsic to the crude extracts of several enzymes, particularly those of plant and microbial origins (Liu et al., 2021). The efficacy of the fungal crude extract as a substitute for commercial rennet can be increased by improving the production process and incorporating purification steps to eliminate nonspecific proteolytic enzymes and other contaminants, thereby augmenting the extract quality.

Furthermore, the effect of moisture and fat contents can be significantly influential on cheese yield. Fat, acting as a relatively inert filler in the coagulum, physically hinders syneresis when included in the curd, thereby affecting moisture retention in the curd. The moisture preserved in the curd directly contributes to the cheese yield and also indirectly, as it carries soluble whey components such as whey proteins, caseinomacropeptide, lactate, and soluble milk salts (Banks, 2007). Thus, the levels of fat and moisture content in fromage frais, produced by the commercial rennet, contributed to an increment in cheese yield compared to samples produced by the crude extract of *M. circinelloides*.

#### Microbiological characteristics

All the samples analyzed exhibited notably low total coliform contamination (less than 10 CFU/g), aligning with the Algerian regulations pertaining to fresh cheese (OJAR n° 35, 1998). The presence of these germs in the sample prepared with the crude extract of *M. circinelloides* is likely attributable to contamination from dairy equipment and utensils used during the cheese-making process.

The search for pathogenic flora, namely *S. aureus* and *Salmonella* spp., in fresh cheeses yielded negative results across all samples. Generally, the absence of these germs emanates from the utilization of raw materials of good microbiological quality and compliance with hygiene rules during cheese preparation. The results of the microbiological analysis of the elaborated fresh cheeses are summarized in Table 4.

**Table 4:** Microbiological quality of the produced fresh cheeses

	Total coliforms [CFU/g]	Faecal coliforms [CFU/g]	*S. aureus* [CFU/g]	*Salmonella* spp. [CFU in 25 g]
Sanitary standards ^(a, b)^	<10	<1	<10	Abs
Cheese produced by				
CHY-MAX^®^ (control)	Abs	Abs	Abs	Abs
Crude extract of *M. circinelloides*	3	Abs	Abs	Abs

a OJAR n°35 (1998),

b Meunier-Godick, (2004), Abs – absence, CFU – Colony Forming Unit

#### Sensory analysis

The majority of tasters polled during the sensory evaluation considered the fresh cheese produced to be of good quality. No essential organoleptic difference was observed in the cheeses produced. Importantly, no bitter taste was detected in any of the cheese samples. They were characterized by a milky-white color and a good spreading ability. Both the surface and internal texture were smooth, devoid of holes, and rindless. The flavor was clean and mildly acidic.

## Conclusion

The maximum production of an acid protease by *M. circinelloides* MG603064.1, grown in SCW, was achieved by incorporating beef extract (0.5%, w/v) and KH_2_PO_4_ (0.4%, w/v). The medium was adjusted to a pH of 5.5, inoculated with an inoculum size of 11% (v/v), and then incubated at 30°C for 72 h. Under these conditions, the milk-clotting activity and the protease activity reached 82.76 ± 2.85 SU/ml and 21.76 ± 1.02 U/ml, respectively.

The biochemical study of the enzyme in the crude extract showed that its aptitude for coagulating milk was close to that of rennin (chymosin) in terms of the effect of pH. However, this enzyme was more sensitive to calcium ions and temperature variations, with optimum values of 25 mM CaCl_2_ and 60°C, respectively. Sonication of the crude extract with a fixed frequency of 35 kHz significantly improved its MCA, where the maximum (107.62 ± 0.00 SU/ml) was achieved after 10 min.

The possibility of substituting commercial rennet with the MCE of *M. circinelloides* in the production of fromage frais from cow’s milk was investigated. Comparative manufacturing tests were carried out with the two enzymatic products. The cheeses produced were analyzed and compared at an organoleptic level; the yields of the transformation from milk to cheese were also specified. Analysis of the experimental data did not reveal significant differences between the use of the two coagulant enzymes. Therefore, the fungal protease of *M. circinelloides* can successfully replace the usual rennet in the manufacture of fresh cheese.

This study proposed the production of an acid protease with an efficient ability to clot milk under conditions close to rennet. Hence, it can be considered a promising substitute in the manufacture of fromage frais. In addition, the production of this enzyme in SCW ensures the reduction of production costs and problems related to environmental pollution. Moreover, it represents an effective means for the valorization of whey. More efficient optimization, purification, and cheese production trials are envisaged using the produced enzymatic extracts.

## Conflict of interest

The authors declare no conflict of interest.
